# Gene expression profile of circulating tumor cells in breast cancer by RT-qPCR

**DOI:** 10.1186/1471-2407-11-422

**Published:** 2011-10-04

**Authors:** Areti Strati, Athina Markou, Cleo Parisi, Eleni Politaki, Dimitris Mavroudis, Vasilis Georgoulias, Evi Lianidou

**Affiliations:** 1 Department of Chemistry, University of Athens, University Campus, 15771, Athens, Greece; 2 Department of Medical Oncology, University of Crete, 71110, Crete, Greece

## Abstract

**Background:**

Circulating tumor cells (CTCs) have been associated with prognosis especially in breast cancer and have been proposed as a liquid biopsy for repeated follow up examinations. Molecular characterization of CTCs is difficult to address since they are very rare and the amount of available sample is very limited.

**Methods:**

We quantified by RT-qPCR *CK-19, MAGE-A3, HER-2, TWIST1, hTERT α+β+*, and *mammaglobin *gene transcripts in immunomagnetically positively selected CTCs from 92 breast cancer patients, and 28 healthy individuals. We also compared our results with the CellSearch system in 33 of these patients with early breast cancer.

**Results:**

RT-qPCR is highly sensitive and specific and can detect the expression of each individual gene at the one cell level. None of the genes tested was detected in the group of healthy donors. In 66 operable breast cancer patients, *CK-19 *was detected in 42.4%, *HER-2 *in 13.6%, *MAGE-A3 *in 21.2%, *hMAM *in 13.6%, *TWIST-1 *in 42.4%, and *hTERT α+β+ *in 10.2%. In 26 patients with verified metastasis, *CK-19 *was detected in 53.8%, *HER-2 *in 19.2%, *MAGE-A3 *in 15.4%, *hMAM *in 30.8%, *TWIST-1 *in 38.5% and *hTERT *α^+^β^+^in 19.2%. Our preliminary data on the comparison between RT-qPCR and CellSearch in 33 early breast cancer patients showed that RT-qPCR gives more positive results in respect to CellSearch.

**Conclusions:**

Molecular characterization of CTCs has revealed a remarkable heterogeneity of gene expression between breast cancer patients. In a small percentage of patients, CTCs were positive for all six genes tested, while in some patients only one of these genes was expressed. The clinical significance of these findings in early breast cancer remains to be elucidated when the clinical outcome for these patients is known.

## Background

Metastasis is a multi-stage process [1] that selects for Circulating Tumor Cells (CTCs) that can infiltrate, survive in and colonize distant organs [2]. Recent advances in this field are supportive for the early dissemination model of metastasis, through the observation that Disseminated Tumour Cells (DTCs) isolated from bone marrow or lymph nodes display disparate changes on all levels of genomic resolution as compared to primary tumor cells [3]. Cancer cell dissemination may be followed by a dormancy period before relapse in one or more organs [4]. Research on DTCs and CTCs present nowadays a challenge, as these cells are well defined targets for understanding tumour biology and tumour cell dissemination in cancer patients [5], and will open new avenues for the early detection of metastatic spread and its successful treatment.

CTCs have been of interest to the medical and research communities for over a century [6]. Data from European groups have sustained the prognostic impact of DTCs in the BM of breast cancer patients [7]. However, sequential peripheral blood analysis is more convenient than BM analyses in patients with solid tumors. CTCs detection and enumeration in breast cancer has been established in several clinical studies, showing a correlation with decreased progression-free survival and overall survival in operable [8-12] and advanced breast cancer [13,14]. Our group has previously shown that the detection of CTCs in peripheral blood of early breast cancer patients before and after chemotherapy through the epithelial molecular marker Cytokeratin-19 (*CK-19*) is of prognostic significance [8-12]. We have recently shown that the detection of CTCs post-chemotherapy in breast cancer patients is associated with involvement of more than three axillary lymph nodes with significantly increased clinical relapses and disease-related deaths [15]. Enumeration and molecular characterization of CTCs can be used as a liquid biopsy for repeated follow up examinations in a variety of human cancers [16-18] and may play a major role in helping to guide targeted therapy [16-20].

Recently, the phenotypical and functional variety of breast cancer cells in primary tumors as well as in DTCs is shown for recognized prognostic factors, such as *HER-2/neu *[19-21], *ER*, *PR *[21] and cancer stem cell markers such as *CD44*, *CD24 *or *ALDH1 *[22,23]. Further molecular characterization of CTCs is important not only to confirm their malignant origin but also to identify diagnostically and therapeutically relevant targets to help stratifying cancer patients for individual therapies [18].

CTCs are rare, comprising a few cells per 10^6 ^hematologic cells in blood of patients with metastasis; hence their isolation presents a tremendous technical challenge [24-26]. DTCs and CTCs can now be detected and characterized at the single cell level [27]. Recent technical advancements in the detection and characterization of CTCs include highly sensitive RT-qPCR [28-30], image-based immunologic approaches like the FDA approved CellSearch system [31], or a combination of molecular and imaging methods [32]. Lately a membrane microfilter device for single stage capture and electrolysis of circulating tumor cells [33] as well as a CTCs microchip were developed [34]. Multimarker RT-PCR can increase sensitivity and specificity of CTCs detection [11,23,26]. By using a multi-marker assay in CTCs in early breast cancer, we have shown that *CK-19*, *Mammaglobin*, and *HER-2 *positive CTCs are associated with shorter disease free survival [11]. Recently, *EpCAM, MUC-1 *and *HER2 *transcripts were detected in CTCs and a major proportion of CTCs in metastatic breast cancer patients showed EMT and tumor stem cell characteristics [23].

The purpose of this study was to quantify by RT-qPCR *CK-19, MAGE-A3, HER-2, TWIST1, hTERT α+β+*, and *mammaglobin *gene transcripts in immunomagnetically positively selected CTCs. We analyzed CTCs from 92 breast cancer patients and 28 healthy individuals and compared our results with the CellSearch system in 33 of these patients with early breast cancer.

## Methods

### Cell lines

The human mammary carcinoma cell lines SKBR-3, MDA-MB-231 and MCF-7 were used for the development of the assay and the generation of gene specific quantification calibrators. Cells were counted in a hemocytometer and their viability was assessed by trypan blue dye exclusion. Serial dilutions of a known number of cells corresponding to 1-1000 cells, for which total RNA isolation and cDNA synthesis was performed, were prepared. These cDNAs were kept in aliquots at -20°C and used for the validation of the assay, prior to the analysis of patient's samples.

### Patients

A total of 92 consecutive patients with breast cancer were studied, 66 patients with stage I-III operable breast cancer at least 2 weeks after the removal of the primary tumor and before the initiation of adjuvant chemotherapy and 26 patients with verified metastasis. A group of 28 healthy female blood donors were used as control. For every patient peripheral blood (20 mL in EDTA) was obtained as previously described [28,29]. All patients signed an informed consent to participate in the study which was approved by the Ethics and Scientific Committees of our Institution.

### RNA extraction-mRNA purification

**Figure 1:**
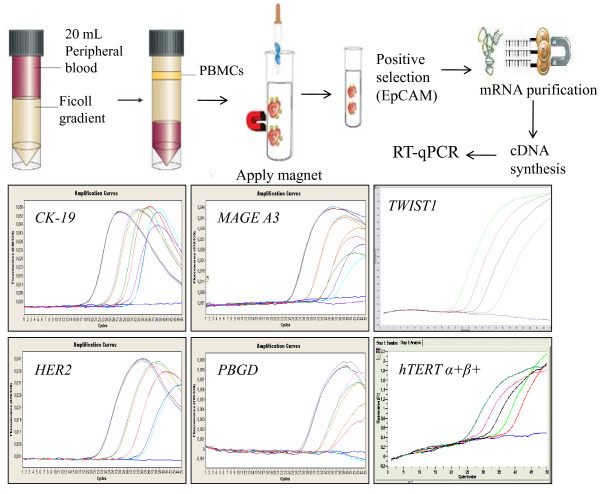
**Schematic diagram of the entire analytical procedure**.

The entire RT-qPCR assay procedure for gene expression in CTCs is outlined in Figure 1.

After dilution of peripheral blood with 20 mL phosphate buffered saline (PBS, pH 7.3), peripheral blood mononuclear cells (PBMCs) were obtained by gradient density centrifugation using Ficol-PaqueTM PLUS (GE Healthcare, Bio-Sciences AB) at 670 g for 30 min at room temperature. The interface cells were removed, washed twice with 40 mL of sterile PBS (pH 7.3, 4°C), at 530 g for 10 min, and resuspended in 1 mL of PBS. Immunomagnetic Ber-EP4 coated capture beads (Dynabeads^® ^Epithelial Enrich, Invitrogen) were used to enrich for epithelial cells, according to manufacturer's instructions. We kept for each sample both isolated fractions: the CTC and corresponding PBMC fraction. Total RNA isolation was performed with TRIZOL (Invitrogen, USA). All RNA preparation and handling steps took place in a laminar flow hood, under RNAse-free conditions. The isolated RNA from each fraction was dissolved in 20 μL of RNA storage buffer (Ambion, USA) and stored at -70°C until used. RNA concentration was determined by absorbance readings at 260 nm using the Nanodrop-1000 spectophotometer (NanoDrop, Technologies, USA). mRNA was isolated from the total RNA, using the Dynabeads mRNA Purification kit (Invitrogen, USA) according to the manufacturer's instructions. cDNA synthesis was performed using the SuperScript™ First-Strand Synthesis System (Invitrogen, USA) in a total volume of 20 μL according to the manufacturer's instructions.

### RT-qPCR

#### Multiplex RT-qPCR

Multiplex RT-qPCR was performed for *CK-19, HER-2, MAGE A3 and PBGD*. Primers and dual hybridization probes were de novo *in-silico *designed (Table 1). Only for *CK-19 *we used the primers and probes previously reported [29], while for *HER-2*, we used the forward primer previously reported [20]. *In-silico *design was performed by using Primer Premier 5.0 software (Premier Biosoft, CA, USA). All primers and probes were designed to match the assay conditions, such as amplicon sizes, and melting temperatures. The specificity of all primer and hybridization probe sequences was first tested by homology searches in the nucleotide database (NCBI, nucleotide BLAST). Each probe set included a 3'-fluorescein (F) donor probe and a 5'- LC acceptor probe that was different for each gene set: *CK-19 *at 640 nm, *MAGE A3 *at 610 nm, *HER-2 *at 670 nm, *PBGD *at 705 nm. A color compensation test was performed by using pure dye spectra so that spectral overlap between dyes was corrected [35]. All oligonucleotides were synthesized at FORTH, Heraklion, Crete, and dual hybridization probes were obtained from TIB MOL, Germany. Multiplex RT-qPCR reactions were performed in the LightCycler 2.0 (Roche, Germany). The amplification reaction mixture (total volume of 10 μL) contained 1 μL of the PCR Synthesis Buffer (10Χ), 1.25 μL of MgCl_2 _(50 mM), 0.4 μL dNTPs (10 mM), 0.8 μL BSA (10 μg/μL), 0.4 μL Hot Start DNA polymerase (Platinum, 5 U/μL, Invitrogen, USA), 1 μL of a mixture containing all eight primers (10 μΜ for each), 0.5 μL of a mixture containing all eight dual hybridization probes (4 μM for each) and DEPC-H_2_O (added to the final volume). Cycling conditions: 95°C/3 min; 45 cycles of 95°C/10 s, annealing at 60°C/60 s and extension at 72°C/30 s.

**Table 1:** Oligonucleotide primers and probes

Target gene	Primer or probe	Sequence (5'-3')	Tm (°C)	Amplicon Size (bp)	Accession **No**.
***CK-19***	Forward	CGG GAC AAG ATT CTT GGT	52.4	142	Y00503
	Reverse	CGT TGA TGT CGG CCT CCA	60.4		
	Probe	TGT CCT GCA GAT CGA CAA CGC CC-FL	71.0		
		LCRed640-CTG GCT GC AGA TGA CTT CCG AAC C-PH	69.0		
***MAGE A3***	Forward	TCG GTG AGG AGG CAA GGT T	60.1	147	NM_005362
	Reverse	GAT GAC TCT GGT CAG GGC AA	57.8		
	Probe	GGA GGA GCA CTG AAG GAG AAG ATC TGC-FL	69.2		
		LC610-AGT GGG TCT CCA TTG CCC AGC TC-PH	68.4		
***HER-2***	Forward	AGC CGC GAG CAC CCA AGT	63.6	188	M11730
	Reverse	CCT GCA CCT CCT GGA TAT CCT G	63.3		
	Probe	CAC CTA CCT GCC CAC CAA TGC CA-FL	70.6		
		LC670-CTG TCC TTC CTG CAG GAT ATC CAG GA-PH	68.8		
***PBGD***	Forward	CCT GAG GCA CCT GGA AGG AG	62.5	163	NM_000190
	Reverse	ATC TTC ATG CTG GGC AGG GA	61.8		
	Probe	TGT GCC AGT AGC CGT GCA TAC AGC-FL	69.1		
		LC705-GAA GGA TGG GCA ACT GTA CCT GAC TG-PH	67.3		
***hTERT ***	Forward	TCA AGG TGG ATG TGA CGG G	59.2	347	AF015950
	Reverse	GGA CTT GCC CCT GAT GCG	61.7		
	Probe	6FAM - CGT GTT CTG GGG TTT GAT GAT GCT GGC GA - TMR	74,5		
***hMAM***	Forward	ACG GAT GAA ACT CTG AGC AAT G	59.4	106	U33147
	Reverse	CAG TTC TGT GAG CCA AAG GT	54.9		
	Probe	6FAM-TGA GGT GTT TAT GCA ATT AAT ATA TGA CAG CAG TC -XT- -PH	64.2		
***TWIST1***	Forward	GGC CGG AGA CCT AGA TGT	54,2	150	NM_000474
	Reverse	CAC GCC CTG TTT CTT TGA AT	57,8		
	Probe	6FAM-TGG ACA GTC TAG AGA CT C TGG AGC TGG-BBQ	66,6		

#### Single RT-qPCR

Single RT-qPCR was performed for *hTERT *α^+^β^+^, *TWIST1 *and mammaglobin. For *hTERT *α^+^β^+ ^we used the same primers and probe and for *mammaglobin *the same primers as previously reported [36,37], while we designed novel hydrolysis probe and primers for *TWIST1 *and a novel hydrolysis probe for *Mammaglobin *(Table 1). The amplification reaction mixture (total volume of 10 μL) contained 1 μL of the PCR Synthesis Buffer (10Χ), 0.5 μL of MgCl_2 _(50 mM), 0.2 μL dNTPs (10 mM), 0.15 μL BSA (10 μg/μL), 0.1 μL Hot Start DNA polymerase (Platinum, 5 U/μL, Invitrogen, USA), 0.5 μL of each primer (10 μΜ), 1.0 μL of each hydrolysis probe (3 μM) and DEPC-H_2_O (added to the final volume). Incubation conditions: 95°C/3 min; 45 cycles of 95°C/10 sec, annealing at 63°C/20 sec for *TWIST1*, 65°C/20 sec for *hTERT α+β+ *[36], 55°C/20 sec for *Mammaglobin *and extension at 72°C/20 sec.

To ensure that amplifiable material was present in all specimens and to avoid false-negative results, real-time amplification of the reference gene *PBGD *was performed for all samples. To reduce the risk of contamination, each procedure such as RNA extraction, cDNA synthesis, preparation of the RT-qPCR reactions and thermocycling, were performed in separate rooms while preparation of the cDNA and PCR reactions were set up in different PCR-hoods. A positive (cell line cDNA) and a negative control (H_2_O) were included in all runs.

### Preparation of RT-qPCR quantification calibrators

For the development and analytical evaluation of the assay, we generated individual PCR amplicons corresponding to the gene-targets studied that would serve as quantification calibrators [36]. For this purpose, total RNA was extracted from SKBR-3, MDA-MB-231 and MCF-7 cells; cDNA was synthesized and served as a template for the amplification of each target of interest by the above described RT-qPCR. PCR products were purified using PureLink™ PCR Purification Kit (Nitrogen, USA) and amplicons for each gene were quantified in the Nanodrop-1000 spectophotometer (NanoDrop, Technologies, USA). Concentrations were converted to copies/μL by use of the Avogadro constant and the molecular weight of each amplicon number of bases of the PCR product multiplied by the mean molecular weight of a pair of nucleic acids which is 660 [38]. A standard stock solution containing all amplicons corresponding to 10^10^copies/μL for each gene transcript was prepared. Serial dilutions of this stock amplicon solution in DNase/RNase-free water ranging from 10^5^copies/μL to 10 copies/μL served as quantification calibrators throughout the study. For quantification of each gene transcript, an external calibration curve was obtained by plotting the concentration of each quantification calibrator expressed as copies/μL vs corresponding quantification cycle (Cq).

### Cell Search

We enumerated CTCs using the CellSearch System (Veridex, USA) in peripheral blood samples of 33 patients with early breast cancer for which an extra volume of peripheral blood was available. 23 mL of blood were collected in CellSave Preservative Tubes (Veridex, USA). In brief, 16.5 mL of whole blood was centrifuged for 10 min at 800 × g and plasma was aspirated. 10 mL of dilution buffer was added and the total blood mixture was layered on 5 mL of Histopaque -1083 and centrifuged for 10 min at 400 × g. Buffer was aspirated and the buffy coat was collected and added in 6.5 mL of total blood. Centrifugation at 800 × g for 10 min followed and the sample was processed into the CellTracks Autoprep System where the Cell Search Circulating Tumor Cell Kit was used (Veridex Warren, NJ.) according to manufacturer's instructions. In brief, this kit contains ferrofluid particles coated with anti-EpCAM antibodies, phycoerythrin conjugated anti-CK antibodies recognizing cytokeratins (8, 18 and/or 19) to specifically identify epithelial cells and allophycocyanin-conjugated anti-CD45 antibody in order to identify white blood cells. Nuclear dye (4',6-diamidino-2-phenylindole/DAPI) was also added so as to fluorescently label the cell nuclei. In the final processing step, the selected cells were transferred automatically to a cartridge in a MagNest cell presentation device after an incubation of at least 20 min in the dark at room temperature. The MagNest was then moved to Cell Tracks Analyzer II, which contains a semi-automated fluorescent microscope (4 fluorescent filter cubes) which captures images of fluorescently labeled cells that are immunomagnetically selected and aligned, covering the entire surface of the cartridge. The images are presented in a gallery format to the operator which classifies according to predetermined criteria (specified by Veridex) for the presence of CTCs. A cell is classified as epithelial cell (CTC) if it meets the following: Nearly round to oval morphology, visible nucleus within the cytoplasm, cytokeratin-phycoerythrin positive, DAPI positive, CD45-allophycocyanin negative and size of at least 4 μm.

## Results

### Validation of CTC gene expression RT-qPCR assay

The ability to multiplex greatly expands the power of qPCR analysis. Multiplexing requires the presentation of evidence demonstrating that accurate quantification of multiple targets in a single tube is not impaired, i.e., that assay efficiency and the LOD are the same as when the assays are run in uniplex fashion. This concern is of particular importance when targets of appreciably lower abundance are coamplified with highly abundant targets [39]. For this reason we performed extensive experiments to validate the performance of multiplex RT-qPCR.

#### Specificity

In multiplex RT-qPCR, we checked the specificity of primers and dual hybridization probes both in the presence and absence of each gene target. So, in individual glass capillaries we tested the specificity of all oligonucleotides when only one individual gene target was present as a template. Each primer and dual hybridization probe pair amplifies specifically only the corresponding target amplicon (Additional file 1 Figure S1). None of the primers and dual hybridization probes gave any signal for any of the gene target transcripts when five different genomic DNAs (500 ng/μL), were analyzed.

#### Limit of Detection and Linearity

A low detection limit is extremely important for CTC analysis. For this reason, before proceeding to patients' samples, we evaluated the limit of detection of the developed CTC gene expression RT-qPCR assay by using quantification calibrators containing a known number of copies/μL, prepared as described [36,39]. For each gene target a calibration curve was generated using serial dilutions of these standards in triplicate for each concentration, ranging from 10^5^copies/μL to 10 copies/μL and showed linearity over 10^5^copies/μL to 10^2^copies/μL with correlation coefficients larger than 0.99 in all cases, indicating a precise log-linear relationship (Additional file 2 Figure S2). The detection limit, quantification limit, mean slope and intercept of the calibration curve as well as the PCR efficiency expressed as E = [10^-1/slope^] -1 for each gene target are shown in Supplemental data, Table 1. In all cases, the LOD (LOD = 3.3 × SD_st_/slope), was found to correspond to 3 copies/μL while the limit of quantification (LOQ) defined as 3 times the LOD was equal to 10 copies/μL. Before proceeding to patients' samples, we evaluated the performance of the developed assays in the SKBR-3 cell line for all gene targets, except *TWIST-1*, where the MDA-MB-231 cell line and *hTERT α+β+ *where the MCF-7 cell line were used. We performed a dilution study for each gene target using cDNA, corresponding to 1000 cells by using serial dilutions in triplicate for each concentration, ranging from 1000 to 1 cells. In these samples, we checked the quantification cycles of each target gene *CK-19, HER-2, MAGE A3 *and *PBGD*. Our results showed linearity over the entire quantification range (1-1000 cells/μL) and correlation coefficients greater than 0.99 in all cases, indicating a precise log-linear relationship.

#### Precision

Repeatability or intra-assay variance (within-run precision) of the multiplex RT-qPCR, was evaluated by repeatedly analyzing 4 cDNA samples corresponding to 1, 10, 100 and 1000 SKBR-3 cells, in the same assay, in 3 parallel determinations. Intra-assay variance expressed as the SDs of the Cq variance [39] for *CK-19*, ranged from 0.15 to 0.48, while for *MAGE A3 *ranged from 0.22 to 0.58, for *HER-2 *ranged from 0.10 to 0.39, and for *PBGD *ranged from 0.16 to 0.41 (Table 2). Intra-assay variance expressed as within-run CVs of copies/μL ranged for *CK-19*, from 10% to 31%, for *MAGE A3 *from 13% to 33%, for *HER-2 *from 6.2% to 29%, and for *PBGD *from 1.3% to 25% (Table 2). Reproducibility or inter-assay variance (between-run precision) [39] of the multiplex RT-qPCR assay, was evaluated by analyzing the same cDNA sample, representing 100 SKBR-3 cells and kept frozen in aliquots at -20°C, over a period of one month on 5 separate assays performed in 5 different days. Between-run CVs were 21% for *CK-19*, 17% for *MAGE A3*, 24% for *HER-2 *and 19% for *PBGD *(Table 2).

**Table 2:** Intra-assay and inter-assay precision of the multiplex RT-qPCR for *CK-19, MAGE A3, HER2 *and *PBGD*.

**SKBR-3**, Number of cells	**Quantification cycle (Cq)**, Mean Cq (SD)	**Copies**, Mean Copies (SD)	CV%
***CK-19***	***Intra assay precision (n = 3)***		
	
**1**	29.75 (0.15)	218 (± 22)	10
**10**	25.90 (0.17)	3.12 (± 0.34) × 10^3^	11
**100**	21.87 (0.17)	5.07 (± 0.61) × 10^4^	12
**1000**	18.37 (0.48)	5.8 (± 1.8) × 10^5^	31
	
	***Inter assay precision (n = 5)***		
	
**100**	21.70 (0.27)	5.8 (± 1.2) × 10^4^	21

***MAGE A3***	***Intra assay precision (n = 3)***		
	
**1**	34.51 (0.58)	43 (± 14)	33
**10**	30.22 (0.31)	6.0 (± 1.1) × 10^2^	18
**100**	26.58 (0.22)	5.79 (± 0.75) × 10^3^	13
**1000**	23.25 (0.42)	4.7 (± 1.1) × 10^4^	23
	
	***Inter assay precision (n = 5)***		
	
**100**	26.41 (0.21)	5.18 (± 0.87) × 10^2^	17

***HER-2***	***Intra assay precision (n = 3)***		
	
**1**	33.38 (0.39)	14 (± 4)	29
**10**	28.81 (0.10)	321 (± 20)	6.2
**100**	24.72 (0.11)	5.33 (± 0.38) × 10^3^	7.2
**1000**	21.48 (0.37)	5.0 (± 1.2) × 10^4^	24
	
	***Inter assay precision (n = 5)***		
	
**100**	24.43 (0.39)	6.6 (± 1.6) × 10^3^	24

***PBGD***	***Intra assay precision (n = 3)***		
	
**10**	32.43(0.16)	27 (± 3)	11
**100**	29.31(0.020)	227 (± 3)	1.3
**1000**	26.26 (0.41)	19 (± 4.8) × 10^2^	25
	***Inter assay precision (n = 5)***		
	
**100**	29.28 (0.27)	324 (± 60)	19

#### Comparison between single and multiplex RT-qPCR

We evaluated the performance of multiplex RT-qPCR by analyzing in parallel the same cDNAs corresponding to 1-1000 SKBR-3 cells and 26 cDNAs from patients' samples by both single and multiplex assays for *CK-19 *and *PBGD*. A successful RT-qPCR multiplex reaction is achieved when multiplex and single assays performed simultaneously on the same run result in similar quantification cycles (Cq) values for the amplification of a particular gene. The multiplex assay correlates very well with single RT-qPCR for all genes studied when the SKBR3 cell line were used (data not shown), while when clinical samples were tested, the multiplex assay correlates very well with single RT-qPCR for both *CK-19 *(R^2 ^= 0.9931) and *PBGD *(R^2 ^= 0.8312).

### Gene expression profile of CTCs in breast cancer

**Figure 2:**
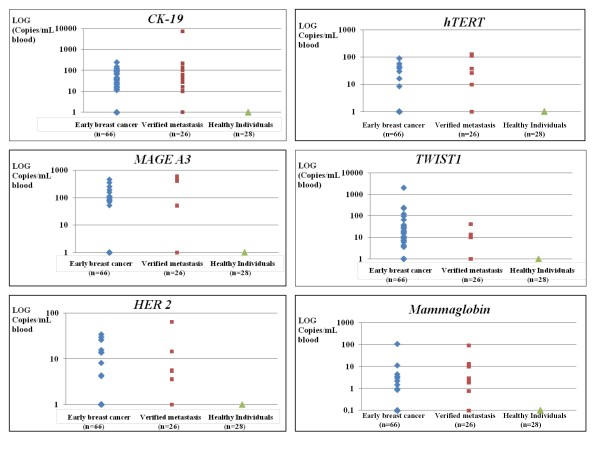
**Quantification of *CK-19, MAGE-A3, HER-2, TWIST-1*, *hMAM *(Mammaglobin), and *h-TERT α+β+ *transcripts in CTCs (copies/mL of peripheral blood)**.

**Figure 3:**
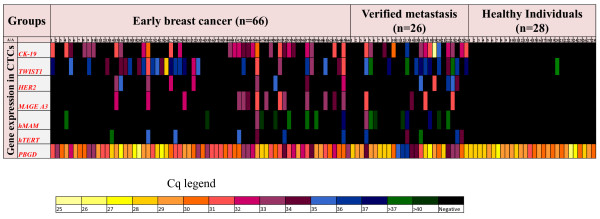
**Heat map of *CK-19, MAGE-A3, HER-2, TWIST-1*, *hMAM *(Mammaglobin), and *hTERT α+β+ *expression in CTCs as quantified by RT-qPCR**.

We quantified *CK-19, MAGE-A3, HER-2, TWIST1, hTERT α+β+*, and *mammaglobin *gene transcripts in immunomagnetically positively selected CTCs from 92 breast cancer patients, and 28 healthy individuals used as a control group. For each patient, we analyzed both the CTC and the corresponding PBMC fraction, used as a negative control. RNA quality of all samples was checked by *PBGD *expression, used as a reference gene. Only samples that were positive for *PBGD *expression were further processed. The expression levels of these genes, expressed as copies/mL of peripheral blood, differed significantly between the healthy normal donors and breast cancer patients, both for the early and the verified metastasis group (Figure 2). We observed a high qualitative and quantitative heterogeneity in the gene expression profile in the CTCs fraction for each individual patient in both groups. As can be seen in Figure 3, in a small percentage of patients (3%), CTCs were positive for all six genes tested, while in some patients only one of these genes was expressed.

#### Healthy individuals

When using Dynabeads we always have a contamination of white blood cells isolated together with CTCs. To overcome this we have also analyzed 28 healthy female volunteers. In this group we did not observe any expression for *CK-19*, *MAGE A3, HER-2*, *hTERT α+β*+, and *Mammaglobin*. For this reason all samples that showed amplification were regarded as positive for these genes. In the same group of healthy individuals we found only one sample with very low expression for TWIST1 (Cq > 37) and have used this Cq as a cut-off for all our samples. According to these results we concluded that contamination of white cells did not affect our expression assays for these markers. PBGD expression that is due to the presence of isolated leucocytes, works as an internal positive control for us since it is giving us the information that isolated RNA is of good quality.

#### Early breast cancer

There was a high heterogeneity in CTC gene expression in early breast cancer patients (Figure 3). There were 22 patients positive for 1 gene (33.3%), 15 patients positive for 2 genes (22.7%), 7 patients positive for 3 genes (10.6%), 2 patients positive for 5 genes (3%), 2 patients positive for all 6 genes studied (3%), while in 18 patients we didn't detect CTCs at all (27.2%). We found 28/66 (42.4%) patients positive for *CK-19*, 14/66 (21.2%) positive for *MAGE-A3*, 9/66 (13.6%) positive for *HER-2*, 28/66 (42.4%) positive for *TWIST1*, 7/66 (10.2%) positive for *hTERT α+β+ *and 9/66 (13.6%) positive for *hMAM*. In early breast cancer, the percentage of double positives for epithelial marker *CK-19 *and mesenchymal marker *TWIST1 *is 14/66 (21.2%). 14 out of 66 patients (21.2%) showed only epithelial characteristics while other 14 patients showed only mesenchymal characteristics. 24 out of 66 patients (36.4%) were negative for both of these markers.

#### Metastatic breast cancer

We observed a high heterogeneity in CTC gene expression in breast cancer patients with verified metastasis (Figure 3). We found 14/26 patients positive for *CK-19 *(53.8%) 4/26 positive for *MAGE-A3 *(15.4%), 5/26 patients positive for *HER-2 *(19.2%), 10/26 (38.5%) positive for *TWIST-1*, 5/26 (19.2%) positive for *hTERT α+β+ *and 8/26 (30.8%) positive for *hMAM*. In this group we found 7 patients positive for 1 gene (26.9%), 4 patients positive for 2 genes (15.4%), 6 patients positive for 3 genes (23.1%), 2 patients positive for 4 genes (7.7%), 1 patient positive for 5 genes (3.85%), while in 6 patients we didn't detect CTCs at all (23.1%).

### Comparison between CellSearch and RT-qPCR in operable breast cancer

**Figure 4:**
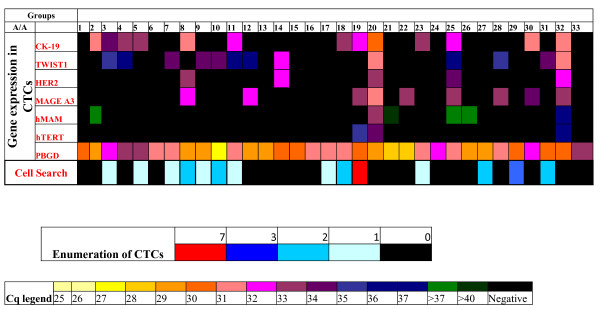
**Comparison of RT-qPCR and CellSearch in early breast cancer patients (n = 33)**.

We analyzed a small number of clinical samples (33 patients with early breast cancer belonging to the group of 66 patients that we analyzed also by RT-qPCR) by both multiplex real time RT-qPCR and the CellSearch System (Veridex, USA). Unfortunately we could only analyze samples for which an extra volume of peripheral blood could be available, since we decided to include this comparison study in the last phase of the recruitment period (Figure 4). The concordance between CellSearch and RT-qPCR for these patients was 18/33 (54.5%). Specifically, 11 patients were found positive and 7 patients were found negative for CTCs by both assays, while 12 patients were found positive by RT-qPCR and negative by CellSearch and 3 patients were found positive by CellSearch and negative by RT-qPCR. Using the CellSearch, 7/33 (21.2%) patients were found to have ≥ 2CTCs/23 mL of peripheral blood and 7/33 (21.2%) patients had one CTC/23 mL of peripheral blood. Using RT-qPCR, 23/33 patients were found positive for at least one gene (69.7%): 13/33 (39.4%) patients were positive for *CK-19*, 9/33 (27.3%) patients were positive for *MAGE-A3*, 5/33 (15.1%) patients were positive for *HER-2*, 3/33 (9.1%) patients were positive for *hTERT α+β+*, 13/33 (39.4%) patients were positive for *TWIST-1 *and 6/33 (18.2%) patients were positive for mammaglobin. In the group of 14/33 (42.4%) patients that were found positive for CTCs by the Cell Search, 11/33 were also positive for at least one gene by RT-qPCR (78.6%) and 3 patients were negative (21.4%). Among the 19/33 patients that were found negative for CTCs by CellSearch, 12 were positive for at least 1 gene (63.2%) and 7/33 patients were negative (36.8%) by RT-qPCR. Patients who tested positive for CTCs, by either CellSearch or gene expression profile, were found to express TWIST1 transcript compared to patients with a negative CTC test by both methods (53.8% vs. 0%; P = 0.013, Fisher's exact test).

## Discussion

Nowadays there is an urgent need for biomarkers for real-time personalized monitoring of the efficacy of systemic adjuvant therapy. At present, the success or failure of anti-cancer therapies is only assessed retrospectively by the absence or presence of overt metastases during the post-operative follow-up period. However, overt metastases are, in general, incurable by most current therapies. Monitoring of CTCs can provide new insights into the clonal selection of resistant tumor cells under biological therapies. The CellSearch is the only US Food and Drug Administration (FDA) approved diagnostic test to automate the detection and enumeration of CTCs, for monitoring disease progression and therapy efficacy in the metastatic prostate, colorectal and breast cancer [13,14,16,18].

However, in addition to enumeration, molecular characterization of CTCs is very interesting to understand their biology and discover an association between gene expression profiles and clinical outcome. Molecular characterization of CTCs is important not only to confirm their malignant origin but also to identify diagnostically and therapeutically relevant targets to help stratifying cancer patients for individual therapies [30]. Molecular characterization of CTCs can expand our knowledge of basic molecular pathways of invasion, migration and immune surveillance and might contribute to the identification of metastatic stem cells with important implications for the development of improved therapies in the near future [40]. Assessing the presence of target antigens on CTCs could be considered as a real-time biopsy allowing the possibility to evaluate the change in tumour phenotype during the clinical course of the disease. A combination of imaging and highly sensitive multi-parametric molecular methods has been very recently evaluated for the molecular characterization of CTCs [41]. However, the molecular characterization of CTCs has been hindered by the very limited amount of available sample.

By using a combination of multiplex and single RT-qPCR we quantitatively evaluated the expression profile of six genes in CTCs isolated from peripheral blood of early and advanced breast cancer patients: a) *CK-19*, an epithelial biomarker of prognostic significance in early breast cancer [8-12,28,29], b) *HER-2*, the therapeutic target of Trastuzumab (Herceptin) whose expression status in metastatic sites differs from that of the original primary tumor in about 5-20% of cases while there is evidence that nearly one third of patients whose primary tumors are HER-2/neu negative might have amplified HER-2/neu on CTCs [19,20], c) *MAGE-A3 *which was found to be expressed in 13% of breast cancer patients and correlated significantly with tumor size and AJCC stage [42], d) *hTERT α+β+*, which is critical for the activation of telomerase [36], e) *hMAM *(*mammaglobin*), a breast tissue specific gene of prognostic value in CTCs [11,37], f) and *TWIST-1*, an epithelial-mesenchymal marker which was found to be expressed in 42% in the CTC (+) group of breast cancer patients by the AdnaTest [23].

Our results show that these genes are specifically expressed in the CTC fraction and not in the corresponding isolated fraction from healthy individuals. A very high percentage of CTC positivity for the expression of at least one gene was found both in early breast cancer (72.8%) and metastasis (76.9%). A remarkable heterogeneity of gene expression was observed for each individual breast cancer patient, since in a small percentage of patients CTCs were positive for all six genes tested, while in some patients only one of these genes was expressed. The clinical significance of these findings in early breast cancer remains to be elucidated when the clinical outcome for these patients is known. More than 50% of patients with verified metastasis had ≥ 2 of these genes expressed. The percentage of CTC positive samples and the Circulating Tumor Load (expressed as copies/mL blood) was higher in the group of patients with verified metastasis, in respect to patients with early breast cancer. *HER-2*, *mammaglobin *and *hTERT α+β+ *were expressed at very low levels, when compared to *CK-19, TWIST-1 *and *MAGE A3*. According to the very small number of gene transcripts detected for each gene target, we can estimate that the number of CTCs/mL of peripheral blood in early breast cancer is very low. Our results are in concordance with the AdnaTest [23], reporting a 42% positivity rate for *TWIST1 *and a 13% positivity rate for *HER2*.

Our preliminary results on the comparison of RT-qPCR with the CellSearch in the adjuvant setting for a limited number of patients, have shown a higher positivity rate in favor of RT-qPCR, despite the fact that we have used the same amount of peripheral blood and positive immunomagnetic selection through EpCAM. This could be possibly explained by the fact that we are additionally isolating mRNA through oligo-dT beads and thus we are using the whole isolated mRNA fraction for cDNA synthesis, in combination with the superior sensitivity of RT-qPCR, as verified at the 10 copies/μL level. To elucidate this, we plan to compare RT-qPCR and CellSearch in the adjuvant setting for a large number of patients in the near future. Consistent with our findings, in a recent study it was reported that there was no association in the detection rates of CTCs from patients with breast cancer when the same samples were analyzed by both the CellSearch and the AdnaTest: 18.4% of patients with primary breast cancer were positive by CellSearch and 35.7% by the AdnaTest [43].

The main limitation of the molecular methods in comparison to the imaging approaches (CellSearch), is the fact that they cannot provide information at the single cell level, eg whether the same CTC is expressing all target genes studied or whether more than one CTC is present, each expressing a different target gene. However, molecular methods such as RT-qPCR in comparison to the imaging approaches, have the potential of giving information for the presence of multiple targets in CTCs, are characterized by high sensitivity and specificity, even at the single cell level, while at the same time the expression of gene targets at the mRNA level can only be achieved through the isolation of viable and not apoptotic CTCs [23,28-30]. Moreover RT-qPCR, is performed in a closed tube and high throughput automated format, can be easily intergraded in external quality control programs, is cheaper and very practical for clinical labs that analyze a large number of patient's samples. Further improvements on CTC isolation technologies are needed, while a combination of imaging and highly sensitive multi-parametric molecular methods in large scale predictive biomarker studies will enable the establishment of CTCs in the clinical setting.

## Conclusions

By using a combination of multiplex and single RT-qPCR we quantitatively evaluated the expression profile of *CK-19, MAGE-A3, HER-2, TWIST1, hTERT α+β+*, and *mammaglobin *in CTCs isolated from peripheral blood of early and advanced breast cancer patients. Our study has revealed a remarkable heterogeneity of gene expression between breast cancer patients and shows that these genes are specifically expressed in the CTC fraction and not in the corresponding isolated fraction from healthy individuals. A very high percentage of CTC positivity for the expression of at least one gene was found both in early breast cancer and metastasis. A remarkable heterogeneity of gene expression was observed for each individual breast cancer patient. In a small percentage of patients, CTCs were positive for all six genes tested, while in some patients only one of these genes was expressed. More than 50% of patients with verified metastasis had ≥ 2 of these genes expressed. Our preliminary results on the comparison of RT-qPCR with the CellSearch in the adjuvant setting for a limited number of patients, have shown a higher positivity rate in favor of RT-qPCR, despite the fact that we have used the same amount of peripheral blood and positive immunomagnetic selection through EpCAM. The clinical significance of these findings in early breast cancer remains to be elucidated when the clinical outcome for these patients is known.

## List of abbreviations

BM: bone marrow; *CK-19*: cytokeratin 19; CTCs: circulating tumor cells; DTCs: disseminated tumor cells; EMT: epithelial mesenchymal transition; ER: estrogen receptor; *HER-2*: human epidermal growth factor receptor 2; *h-TERT*: human telomerase reverse transcriptase; LOD: limit of detection; LOQ: limit of quantification; *PBGD*: porphobilinogen deaminase; PR: progesterone receptor; RT-qPCR: Reverse Transcription quantitative Polymerase Chain Reaction; *MAGE-A3*: Melanoma-associated antigen 3

## Competing interests

The authors declare that they have no competing interests.

## Authors' contributions

EL designed, and supervised the project; AS, AM and EL developed the assay; AS and AM performed most of the experiments; CP performed *h-TERT *expression study in CTCs; EP performed the Cell Search measurements in CTCs; VG and DM provided the clinical samples; AS and EL wrote the manuscript; All authors read and approved the final manuscript.

## Pre-publication history

The pre-publication history for this paper can be accessed here:

http://www.biomedcentral.com/1471-2407/11/422/prepub

## Supplementary Material

Additional file 1 **Figure S1. Specificity of multiplex RT-qPCR for *CK-19*, *MAGE A3*, *HER-2 *and *PBGD***. Specificity of primers and dual hybridization probes both in the presence and absence of each gene target. Click here for file

Additional file 2 **Figure S2. Quantification of *CK-19*, *MAGE A3*, *HER-2*, *PBGD *mRNA-positive cells by multiplex RT-qPCR (copies/μL, measured in triplicate)**. Evaluation of the limit of detection of the developed CTC gene expression RT-qPCR assay by using quantification calibrators containing a known number of copies/μL. Click here for file
