# Supplementary figures and images for: Gene expression profile of circulating tumor cells in breast cancer by RT-qPCR
Source: BMC Cancer. 2011 Oct 4;11:422. doi: 10.1186/1471-2407-11-422 (PMC3224356; doi:10.1186/1471-2407-11-422)

## Slide 1
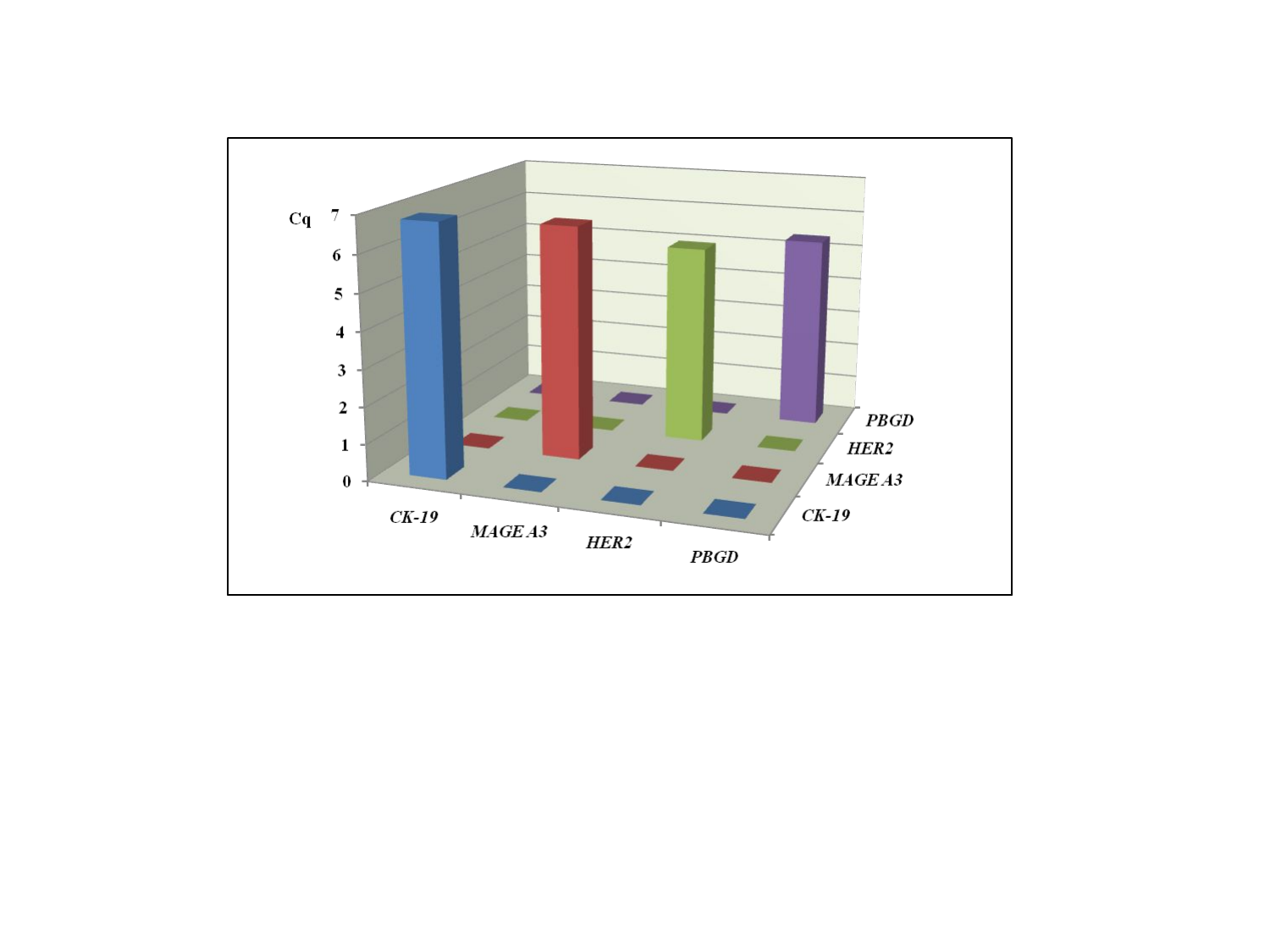

Supplement: Additional file 1 — Figure S1. Specificity of multiplex RT-qPCR for CK-19, MAGE A3, HER-2 and PBGD. Specificity of primers and dual hybridization probes both in the presence and absence of each gene target. [file 1471-2407-11-422-S1.PPT]

## Slide 1
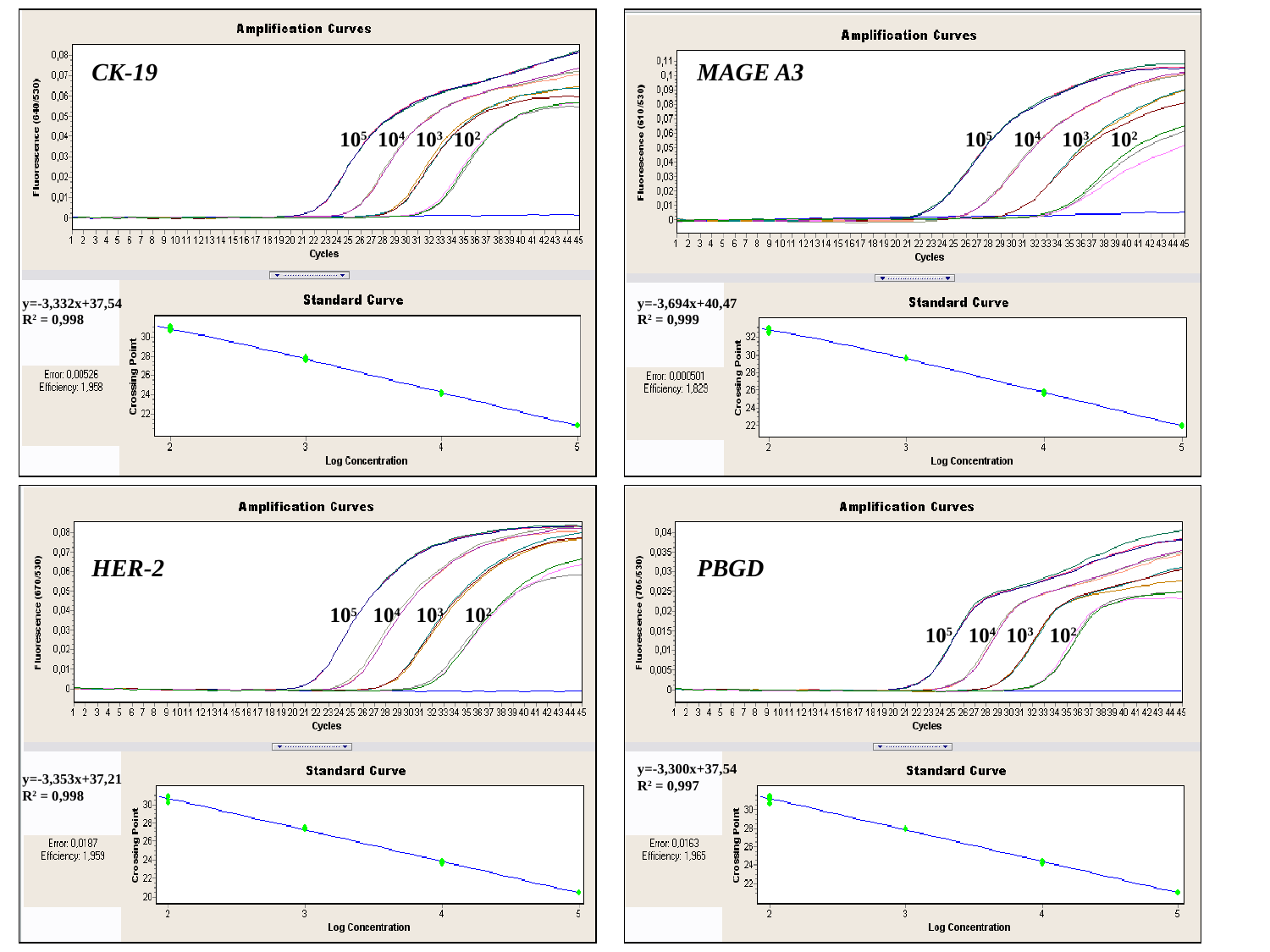

CK-19
MAGE A3
105 104 103 102
105 104 103 102
y=-3,332x+37,54
R2 = 0,998
y=-3,694x+40,47
R2 = 0,999
HER-2
PBGD
105 104 103 102
105 104 103 102
y=-3,300x+37,54
R2 = 0,997
y=-3,353x+37,21
R2 = 0,998

Supplement: Additional file 2 — Figure S2. Quantification of CK-19, MAGE A3, HER-2, PBGD mRNA-positive cells by multiplex RT-qPCR (copies/μL, measured in triplicate). Evaluation of the limit of detection of the developed CTC gene expression RT-qPCR assay by using quantification calibrators containing a known number of copies/μL. [file 1471-2407-11-422-S2.PPT]
